# A multinational, drug utilisation study to investigate the use of dexmedetomidine (DEXDOR®) in clinical practice in the EU

**DOI:** 10.1186/2197-425X-3-S1-A322

**Published:** 2015-10-01

**Authors:** C Garratt, M Weatherall, P Pohjanjousi, R Aantao, G Conti, M Lewis, N Moore, S Perez-Gutthann

**Affiliations:** Orion Pharma, Nottingham, United Kingdom; Orion Pharma, Kuopio, Finland; University of Turku, Turku, Finland; Catholic University of Rome, Rome, Italy; EPES Epidemiology GmbH, Berlin, Germany; Bordeaux Pharmacoepi, Bordeaux, France; RTI Health Solutions, Barcelona, Spain

## Introduction

Dexmedetomidine (Dex) is a sedative drug approved as dexdor® (Orion Pharma, Finland) for ICU sedation in adults in the European Union in 2011. This observational, retrospective drug utilisation study was requested by the Committee for Human Medicinal Products for Human Use of the European Medicines Agency to investigate Dex use in clinical practice.

## Objectives

The objective of the study was to evaluate how Dex is used in the EU, with particular focus on off-label use including the paediatric population.

## Methods

Study countries/sites were chosen by a blinded multi-national, multi-specialist, independent group from those with highest Dex use, based on sales. All patients treated with Dex during the enrollment period were to be included. Anonymised data on demographics, treatment indication, dosing, concomitant medications and treatment effectiveness were collected retrospectively. Informed consent was waived to avoid influence of the study on the prescribing of Dex.

## Results

Data from 2000 patients were collected from 16 hospitals in 4 EU countries (Finland 750, Poland 505, Germany 470, Austria 275) between June 2013 and December 2014. The median age was 62 years (males 70.2%). The proportion of paediatric patients was 5.2% with the highest incidence in Austria and Finland. Dex was primarily used in adult ICU (86.0%) for ICU sedation (78.6%) and mostly dosed according the label. Overall in 84.9% of administrations the intended sedative effect was obtained. The most common clinical situations in the ICU in which Dex was used varied between countries but was predominantly agitation despite existing sedation, delirium and difficult to wean from the ventilator. Other clinical objectives included reducing/replacing other sedatives and facilitating sleep.

## Conclusions

This drug utilisation study indicates that Dex is mostly used according to the terms of its product licence, although a variable degree of use was also seen in other settings and populations.

## Grant Acknowledgment

This study was sponsored by Orion Pharma

